# Genetic diversity among some canola cultivars as revealed by RAPD, SSR and AFLP analyses

**DOI:** 10.1007/s13205-013-0165-x

**Published:** 2013-09-13

**Authors:** Reda E. A. Moghaieb, Etr H. K. Mohammed, Sawsan S. Youssief

**Affiliations:** 1 Department of Genetics and Genetic Engineering Research Center, Faculty of Agriculture, Cairo University, Giza, Egypt; 2 Plant Protection Research Institute, Agriculture Research Center, Ministry of Agriculture, Dokki, Giza, Egypt

**Keywords:** Canola, RAPD, SSR, AFLP, Genetic diversity, Molecular markers

## Abstract

To assess the genetic diversity among four canola cultivars (namely, Serw-3, Serw-4, Misser L-16 and Semu 249), random amplified polymorphic DNA (RAPD), simple sequence repeat polymorphism (SSR) and amplified fragment length polymorphism (AFLP) analyses were performed. The data indicated that all of the three molecular markers gave different levels of polymorphism. A total of 118, 31 and 338 markers that show 61, 67.7 and 81 % polymorphism percentages were resulted from the RAPD, SSR and AFLP analyses, respectively. Based on the data obtained the three markers can be used to differentiate between the four canola cultivars. The genotype-specific markers were determined, 18 out of the 72 polymorphic RAPD markers generated were found to be genotype-specific (25 %). The highest number of RAPD specific markers was scored for Semu 249 (15 markers), while Serw-4 scored two markers. On the other hand, Serw-3 scored one marker. The cultivar Semu 249 scored the highest number of unique AFLP markers, giving 57 unique markers, followed by Misser L-16 which was characterized by 40 unique AFLP markers, then Serw-3 giving 31 unique markers. While Serw-4 was characterized by the lowest number producing 14 unique positive markers. The dendrogram built on the basis of combined data from RAPD, SSR and AFLP analysis represents the genetic distances among the four canola cultivars. Understanding the genetic variability among the current canola cultivars opens up a possibility for developing a molecular genetic map that will lead to the application of marker-assisted selection tools in genetic improvement of canola.

## Introduction

Canola (*Brassica napus* L.) is considered as the most important source of vegetable oil and protein-rich meal worldwide. It was developed through conventional plant breeding from rapeseed. It ranks the third among the oil crops, following palm oil and soya oil and the fifth among economically important crops, following rice, wheat, maize and cotton (Sovero 1993; Stoutjesdijk et al. 2000). There are increased domestic and export market opportunities for canola oil that can be realized through the development of high-oleic acid canola to replace saturated palm oil in food service applications (Spector 1999; Stoutjesdijk et al. 2000). In addition, high-oleic acid oils are more nutritionally beneficial because oleic acid had cholesterol-lowering properties, whereas saturated fatty acids tend to raise blood cholesterol levels (Stoutjesdijk et al. 2000).

Egypt recently experienced a solid decline in the total oilseed production on account of reduced cotton area, which overwhelmed increases in soybean output (Hassan and Sahfique 2010). This increased demand, and the need for crop diversification, will undoubtedly promote increased acreage of canola in Egypt. According to the Egyptian Ministry of Agriculture and Land Reclamation (2003–07), the seed oil content in the canola cultivar Serw-4 riches 42 %, while the cultivar Serw-3 have 40 % therefore these two cultivars seems to be promising for its high oil contents. In Egypt, there are agricultural opportunities to increase canola production by expanding into the new reclaimed regions.

Traditional breeding strategies that have attempted to utilize genetic variation arising from varietal germplasm, induced mutations and somaclonal variations of cell and tissue cultures have met with only limited success (Kebede et al. 2010). Therefore, the methods that evaluate and identify the genotypes more precisely during the growing season, especially at early stages, are preferred by plant breeders (Charcosst and Moreau 2004; Basunanda et al. 2007).

The analysis of genetic variation and relatedness in germplasm are of great value for genetic resources conservation and plant breeding programs to determine the best crosses between different genotypes. Over the years, the methods for assessing genetic diversity have ranged from classical strategies such as morphological analysis to biochemical and molecular techniques (Marijanovic et al. 2009).

In recent years, the identification of *Brassica* cultivars has depended on the application of different DNA markers. As DNA sequences are independent of environmental conditions, identification can be determined at any stage of plant growth (Ahmad et al. 2007; Younessi et al. 2011).

DNA markers reflect directly individual differences at the level of DNA molecules, and cover coding and non-coding regions of the genome (Dandelj et al. 2004). They are not affected by environment, developmental stage, certain tissue and organ, and have high-genomic frequency, high polymorphism and mostly a random genomic distribution (Charcosst et al. 2004; Zeng et al. 2004).

Several molecular techniques have been developed to assess genetic diversity and discriminate between genotypes in different crops. These include restriction fragment length polymorphism (RFLP) (Jaroslava et al. 2002), random amplified polymorphic DNA (RAPD) (Ahmad et al. 2007), amplified fragment length polymorphism (AFLP) (Vos et al. 1995) and microsatellites or simple sequence repeat polymorphism (SSR) (Halton et al. 2002).

The objectives of this investigation were to determine the genetic variability among four canola cultivars (namely Serw-3, Serw-4, Misser L-16 and Semu 249) at the molecular levels using RAPD, SSR and AFLP markers and to use the combined data to construct a phylogenetic tree. The genotype-specific markers were also determined.

## Materials and methods

### Plant materials

Four canola genotypes, namely Serw-3, Serw-4, Misser L-16 and Semu 249 were kindly provided by Field Crop Institute, Agricultural Research Center, Ministry of Agriculture, Egypt.

### RAPD analysis

#### DNA extraction

Genomic DNA was isolated from young leaves of greenhouse grown plants using the CTAB method early described in Rogers and Bendich (1985). The quality and quantity of DNA were determined using agarose gel (0.8 %) electrophoresis and spectrophotometer (Table 1).

#### PCR analysis

PCR reactions were performed in a total volume of 20 μl containing 10 ng DNA, 200 μM dNTPs, 1 μM of 15 arbitrary 10-mer primers (Operon Technology, Inc., Alameda, CA, USA), 0.5 units of Red Hot Taq polymerase (AB gene House, UK) and 10 × Taq polymerase buffer (AB gene House, UK). For DNA amplification Biometra thermal cycler (2720) was programmed as follows: 94 ºC for 5 min followed by 35 cycles 94 ºC for 1 min, 35 ºC for 1 min, 72 °C for 1 min and 72 ºC for 7 min.

The amplification products were analyzed by electrophoresis in 1 % agarose in TAE buffer, stained by ethidium bromide and photographed under UV light. The sequence of the tested primers was as follows:

Simple sequence repeat polymorphism analysis PCR reaction mix includes the following: DNA, 10 ng/μl; 10 × buffer; 10 mM dNTPs; 50 mM MgCl_2_; 10 μM each of forward and reverse primers. The PCR profile starts with 95 °C for 5 min followed by 35 cycles of denaturation at 94 °C for 1 min, annealing at 55 °C for 1 min extension at 72 °C for 2 min. A final extension 72 °C for 7 min was included. The PCR products were electrophoresed in a 2 % agarose gels (for SSRs) at 100 V. The gel was then stained in ethidium bromide for 30 min, and then observed on a UV trans-illuminator.

### AFLP analysis

Amplified fragment length polymorphism was performed as described by Vos et al. (1995) using the GIBCO BRL system I (Cat. No. 10544) according to the manufacturer’s protocol.

Amplified fragment length polymorphism amplification products were separated in a vertical denaturing 6 % polyacrylamide gel in a Sequi-Gen Cell (Bio-Rad Laboratories Inc.) as described by Bassam et al. (1991).

#### Band scoring and cluster analysis

The RAPD, SSR and AFLP gel images were scanned using the Gel Doc 2000 Bio-Rad system and analyzed with Quantity One Software v 4.0.1 (Bio-Rad Laboratories, Hercules, Co. USA). The bands were sized and then binary coded by 1 or 0 for their presence or absence in each genotype. The systat ver. 7 computer program was used to calculate the pairwise differences matrix and plot the dendrogram among canola cultivars (Yang and Quiros 1993). Cluster analysis was based on similarity matrices obtained with the unweighed pair-group method (UPGMA) using the arithmetic average to estimate the dendrogram (Table 2).

## Result and discussion

**Fig. 1:**
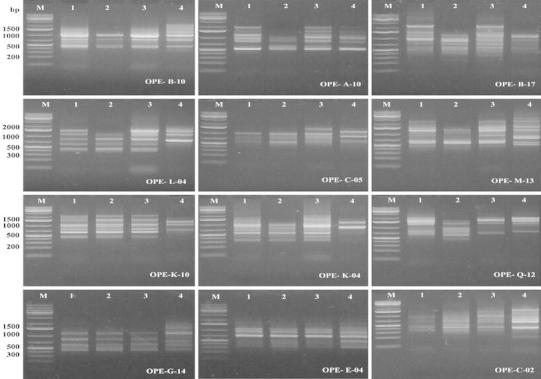
Genetic polymorphism among canola cultivars as revealed by RAPD analysis. M: 1 kbp plus DNA ladder, 1-4: the canola cultivars Serw-3, Serw-4, Misser L-16 and Semu 249, respectively

Recently, the identification of *Brassica* cultivars has depended on the application of different DNA markers. As DNA sequences are independent of environmental conditions, identification can be determined at any stage of plant growth (Ahmad et al. 2007). The RAPD markers are easier and quicker to use and preferred in applications where relationships between closely related breeding lines are of interest. This analysis detects nucleotide sequence polymorphisms in DNA using a single primer of arbitrary nucleotide sequence. DNA profiling with suitable RAPD primers could be used for identification and discrimination between of oilseed rape cultivars (Mailer et al. 1997; Ahmad et al. 2007). In the present work to study the genetic variability among four canola cultivars, RAPD analysis was performed. All the primer tested produced amplification products with variable band number. One hundred eighteen RAPD markers were obtained and out of them 72 were polymorphic (61 %) and can be considered as useful RAPD markers for the four canola cultivars used (Fig. 1; Table 3). The highest number of RAPD bands was recorded for primers OPE-M-13 (11 bands), followed by OPE-Q-14 (10 bands), while the lowest was scored for OPE-C-02 and OPE-G-14 (6 bands).

**Table 1:** Names and sequences of RAPD primers used to assess the genetic variability among the four canola culivar

Primers name	Sequence
OPE-A-10	5′-GTGATCGCAG-3′
OPE-B-10	5′-CTGCTGGGAC-3′
OPE-B-17	5′-AGGGAACGAG-3′
OPE-C-02	5′-GTGAGGCGTC-3′
OPE-C-05	5′-GATGACCGCC-3′
OPE-E-04	5′-GTGACATGCC-3′
OPE-G-14	5′-GGATGAGACC-3′
OPE-K-04	5′-CCGCCCAAAC-3′
OPE-K-15	5′-CTCCTGCCAA-3′
OPE-K-10	5′-GTGCAACGTG-3′
OPE-L-04	5′-GACTGCACAC-3′
OPE-M-13	5′-GGTGGTCAAG-3′
OPE-N-13	5′-AGCGTCACTC-3′
OPE-P-09	5′-GTGGTCCGCA-3′
OPE-Q-14	5′-GGACGCTTCA-3′

**Table 2:** Names and sequences of the SSR loci used to characterize the four canola genotypes

Primer name	Sequence of forward primers	Sequence of reverse primers
RM 206	5′-CCCATGCGTTTAACTATTCT-3′	5′-CGTTCCATCGATCCGTATGG-3′
RM 264	5′-GTTGCGTCCTACTGCTACTTC-3′	5′-GATCCGTGTCGATGATTAGC-3′
RM 561	5′-GAGCTGTTTTGGACTACGGC-3′	5′-GAGTAGCTTTCTCCCACCCC-3′
RM 544	5′-TGTGAGCCTGAGCAATAACG-3′	5′-GAAGCGTGTGATATCGCATG-3′
RM 547	5′-TAGGTTGGCAGACCTTTTCG-3′	5′-GTCAAGATCATTCTCGTAGCG-3′
RM 519	5′-AGAGAGCCCCTAAATTTCCG-3′	5′-AGGTACGCTCACCTGTGGAC-3′
RM 566	5′-ACCCAACTACGATCAGCTCG-3′	5′-CTCCAGGAACACGCTCTTTC-3′

**Table 3:** Total number of scorable bands, polymorphism % and band size of RAPD markers obtained by 15 random primers

Primer	Total scorable bands	Polymorphic bands	Polymorphism ( %)	Band size range
OPE-A-10	7	4	57	500–2,000
OPE-B-10	8	2	25	400–2,000
OPE-B-17	9	4	44	250–2,000
OPE-C-02	6	4	62	500–1,300
OPE-C-05	8	3	37	200–1,500
OPE-E-04	7	1	14	300–1,500
OPE-G-14	6	2	33	450–1,500
OPE-K-04	9	7	77	350–2,000
OPE-K-10	7	3	42	450–2,000
OPE-K-15	5	3	60	600–2,000
OPE-L-04	8	5	62	400–1,500
OPE-M-13	11	8	72	500–2,200
OPE-N-13	8	5	62	350–2,000
OPE-P-09	9	5	55	400–1,400
OPE-Q-14	10	8	80	500–2,000
Total	118	72	61	

The genotype-specific RAPD markers for the different canola cultivars used are listed in Table 4; 18 out of the 72 polymorphic RAPD markers generated were found to be genotype-specific (25 %). The highest number of RAPD specific markers was scored for Semu 249 (15 markers), while Serw-4 scored two markers. On the other hand, Serw-3 scored one marker.

**Table 4:** Canola genotypes and their specific RAPD markers

Genotypes	Markers	Total marker
Serw-3	OPE-B-17,400	1
Serw-4	OPE-C-05,500, OPE-Q-14,500	2
Misser L-16	–	–
Semu 249	OPE-B-10,2000,400,OPE-L-04,700,OPE-C-02,650,OPE-M-13,2200,700,OPE-C-05,200,OPE-G-14,1500,700,OPE-K-04,950,OPE-K-10,1400,OPE-Q-14,1300,OPE-N-13,800,OPE-P-09,1400,500	15
Total		18

Microsatellite or SSR is another PCR-based marker which is preferred by many geneticists and plant breeders because of higher repeatability, co-dominant nature, specificity and having multiple alleles (Cheng et al. 2009). Cruz et al. (2007) employed the SSR markers to characterize the flowering time of spring and winter type *B. napus* L. germplasm. Qu et al. (2012) studied the genetic diversity and performed relationship analysis among the *B. napus* germplasm using SSR markers.

**Fig. 2:**
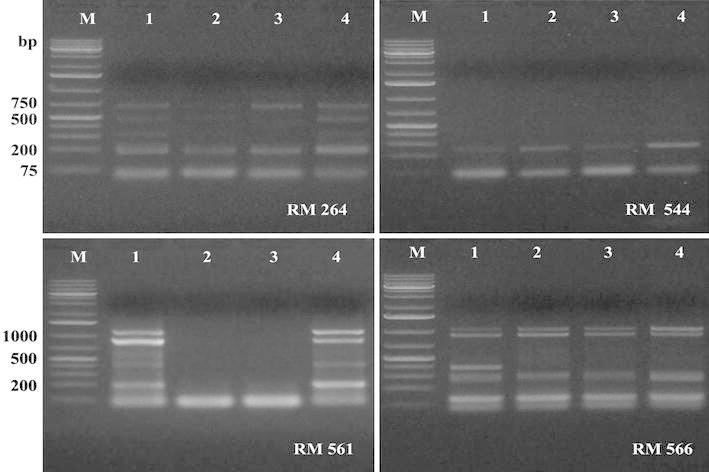
The genetic variability among canola cultivars as revealed by SSR analysis

In the present study, seven SSR primer pairs flanking dinucleotide SSR (GA or AG) were used to investigate the level of polymorphism among the four canola cultivars. The seven SSR primer sets revealed 31 alleles and 21 out of them were polymorphic (67.7 %). All primers showed different levels of polymorphism except RAM 206, RM 544 and RM 519 which showed no polymorphism among the four canola cultivars (Fig. 2; Table 5). The size of the detected alleles produced from using the SSR primer sets ranged from 75–2000 bp, which reflects a large difference in the number of repeats between the different alleles. These results agreed with Moghaddam et al. (2009) that could successfully assessed the genetic diversity in rapeseed cultivars using RAPD and microsatellite markers.

**Table 5:** Total bands, polymorphic bands and polymorphism percentages among canola cultivars as revealed by SSR analysis

Primer name	Total scorable alleles	Polymorphic alleles	Polymorphism (%)
RM 206	2	0	0
RM 264	5	2	40
RM 561	6	5	83
RM 544	2	0	0
RM 547	7	4	57
RM 519	3	0	0
RM 566	6	1	16
Total	31	21	67.7

**Table 6:** Total bands, polymorphic bands and polymorphism percentages among canola cultivars as revealed by AFLP analysis

Primer	Total scorable bands	Polymorphic band	Polymorphism (%)	Band size range	Origin-specific markers
M-CAG/E-ACC	98	78	79	473–979	33
M-CAC/E-AGG	130	117	90	471–983	58
M-CAT/E-AGG	110	101	91	471–1,044	51
Total	338	296	87		142

The AFLP technique has been used for varietal fingerprinting, mapping and genetic diversity studies. The major advantage of the AFLP technique over the other techniques is that it generates a larger number of amplified products in a single reaction (Powell et al. 1996). AFLP’s on average reveal more polymorphic markers then either RFLP or RAPD’s. In *B. napus* Lombard et al. (2000) indicate that two AFLP primer pair combinations were sufficient to distinguish 83 different cultivars from each other.

**Fig. 3:**
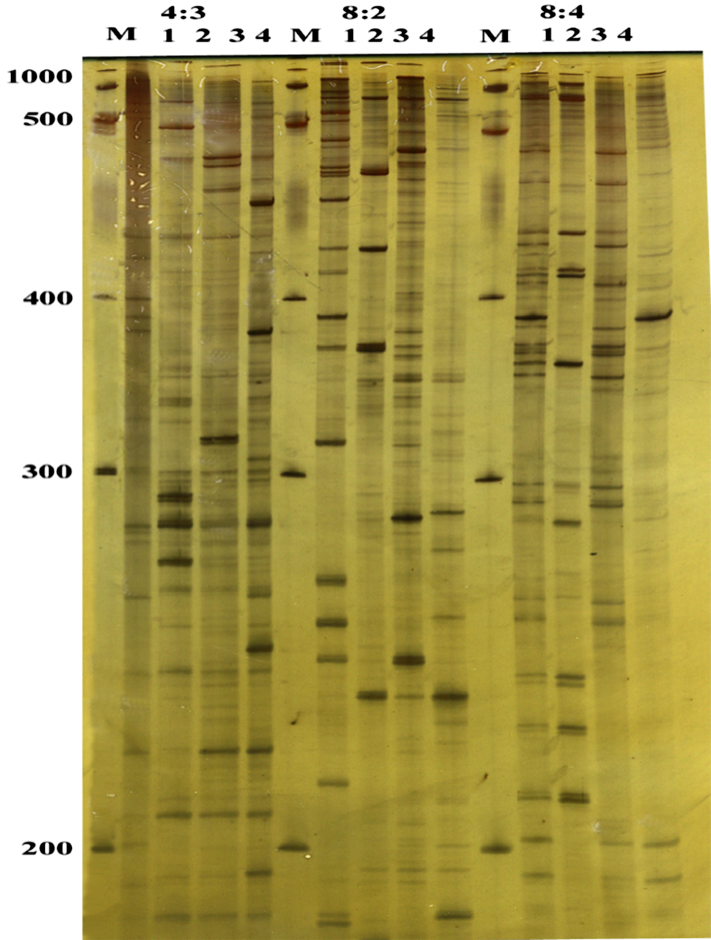
The genetic diversity among canola cultivars as revealed by AFLP analysis

In the present investigation, the AFLP analysis was performed using three selective primer combinations and generated a total of 338 bands (Fig. 3; Table 6) The number of markers observed per primer combination ranged from 98 to 130. The total accounting marker number was 338 amplified bands, representing 87 % polymorphism and an average number of polymorphic bands per AFLP primer combination ranged from 79 to 91 bands. The highest percentage of polymorphism was obtained with M-CAT/E-AGG (91 %) followed by M-CAC/E-AGG (90 %) then M-CAG/E-ACC (79 %). The M-CAC/E-AGG produced the highest polymorphic bands (117), while the lowest number was obtained with M-CAG/E-ACC (78). As shown in Table 6, the size of AFLP fragments generated by the different primer combinations ranged from 471–1044 bp and the polymorphic fragments were distributed across the entire size range (Fig. 3; Table 6).

The unique AFLP markers that characterized the four canola varieties are listed in Table 7. Based on the data obtained, it can be distinguished between the four canola cultivars tested according to the AFLP specific markers. The cultivar Semu 249 exhibited the highest number of unique AFLP markers, giving 57 markers, followed by Misser L-16 which was characterized by 40 unique AFLP markers, then Serw-3 with 31 unique markers. While Serw-4 was characterized by the lowest number producing 14 unique positive markers.

**Table 7:** Canola genotype-specific AFLP markers

Genotypes	Markers	Total marker
Serw-3	M-CAG/E-ACC (931, 890, 824, 561, 553, 509)	31
	M-CAC/E-AGG (954, 947, 943, 915, 664, 542, 536, 533, 525, 522, 472)	
	M-CAT/E-AGG (1040, 978, 958, 928, 887, 821, 811, 604, 591, 567, 565, 533, 530, 522)	
Serw-4	M-CAG/E-ACC (888, 592)	14
	M-CAC/E-AGG (933, 869, 823, 810, 681, 653, 611, 471)	
	M-CAT/E-AGG (974, 876, 633, 527)	
Misser L-16	M-CAG/E-ACC (952, 943, 854, 821, 647, 559)	40
	M-CAC/E-AGG (958, 952, 939, 935, 931, 913, 897, 888, 863, 861, 647, 639, 609, 591, 574, 553, 542, 539, 531)	
	M-CAT/E-AGG (951, 947, 932, 865, 862, 794, 735, 587, 580, 567, 534, 520, 503, 501, 471)	
Semu 249	M-CAG/E-ACC (970, 958, 909, 886, 880, 878, 861, 857, 843, 835, 823, 817, 678, 655, 564, 535, 531, 530, 511)	57
	M-CAC/E-AGG (977, 867, 865, 833, 816, 808, 674, 649, 633, 629, 606, 596, 583, 572, 569, 554, 549, 537, 511, 510)	
	M-CAT/E-AGG (869, 814, 788, 778, 763, 750, 741, 732, 717, 711, 689, 683, 666, 662, 661, 621, 546, 494)	
Total		142

**Fig. 4:**
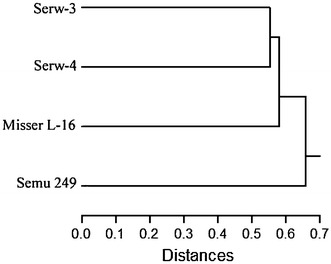
Clustering of four canola cultivars based on pooled RAPD; SSR and AFLP markers

The RAPD; SSR and AFLP based dendrogram group the investigated cultivars into two main clusters. The first cluster included Semu 249, the second cluster is divided into two sub-clusters the first one contains the cultivar Misser L-16 and the second one has the cultivars Serw-3 and Serw-4 (Fig. 4). These results indicate that the cultivars Serw-3 and Serw-4 may possess a high degree of genetic similarity; also their ancestors might be closely related (Fig. 4).

The dendrogram built on the basis of combined data from RAPD, SSR and AFLP analysis represents the genetic distances among the four canola varieties (Fig. 4).

## Conclusion

The results of the present study indicate that DNA markers represent efficient tools for estimating the genetic variability and the genetic relationships among the four canola cultivars. The markers generated are enough to distinguish between the different genotypes used. The genotype-specific molecular markers were determined and these markers can be considered as useful markers for high oil production in canola breeding programs. This opens up a possibility for developing a molecular genetic map that will lead to the application of marker-assisted selection tools in genetic improvement of canola.
